# Weight control efforts and practices and health professional advice: a cross-sectional national survey in England

**DOI:** 10.1136/bmjopen-2024-086764

**Published:** 2024-11-11

**Authors:** Sarah E Jackson, William Warr, Jamie Brown, Jamie Hartmann-Boyce, Susan A Jebb, Kate Tudor, Lion Shahab, Paul Aveyard

**Affiliations:** 1 Department of Behavioural Science and Health, University College London, London, UK; 2 SPECTRUM Consortium, Edinburgh, UK; 3 Nuffield Department of Primary Care Health Sciences, Oxford University, Oxford, UK; 4 Department of Health Promotion and Policy, University of Massachusetts Amherst, Amherst, Massachusetts, USA; 5 NIHR Oxford Biomedical Research Centre, John Radcliffe Hospital, Oxford, UK; 6 Nesta, London, UK; 7 NIHR Oxford Health Biomedical Research Centre, Warneford Hospital, Oxford, UK

**Keywords:** Primary Care, Obesity, Surveys and Questionnaires, PUBLIC HEALTH

## Abstract

**Abstract:**

**Objectives:**

There is evidence that general practitioners (GPs) can increase the uptake of weight management programmes that enhance weight loss compared with self-directed efforts, but the rate at which they do so is unclear. This study examined the prevalence of weight control efforts and practices, the reported frequency and impact of receipt of GP advice on weight loss attempts and perceptions of the appropriateness of health professionals delivering weight loss advice.

**Design:**

A nationally representative cross-sectional survey.

**Setting:**

England.

**Participants:**

1722 adults (≥16 years) surveyed in October 2018 (mean (SD) age=47.4 (19.2), 51.1% women).

**Main outcome measures:**

Weight control efforts and practices, whether a GP gave advice or a specific referral/prescription medication, perception of the appropriateness of GP weight loss advice.

**Results:**

Two-thirds (64.7% (95% CI 58.1% to 71.3%)) of people with obesity reported trying to lose weight. Of people with obesity who visited their GP in the past year, 40% (95% CI 32.2% to 47.7%) recalled receiving any advice on weight loss: 30.8% (95% CI 23.5% to 38.2%) general advice and 9.2% (95% CI 4.6% to 13.7%) a referral to a weight loss service or prescription medication for weight loss. Having received weight loss advice from a GP was strongly associated with a greater likelihood of trying to lose weight (general advice: OR_adj_=4.49, 95% CI 2.52 to 8.00; referral/medication: OR_adj_=9.25, 95% CI 2.65 to 32.3). Views on whether health professionals should deliver weight loss advice were mixed, with a substantial minority (19.4% (95% CI 17.5% to 21.4%)) finding it unacceptable. People with a BMI outside of the healthy weight range (underweight/overweight/obesity), women and those from more disadvantaged social grades were less likely to find it acceptable.

**Conclusions:**

Most people with obesity reported trying to lose weight but less than half recalled receiving advice on weight loss from their GP in the past year and few were referred to community weight-loss programmes. Those who recalled receiving GP advice on weight loss were substantially more likely to report taking action to lose weight. One in five people thought GP advice on weight loss was inappropriate.

**STRENGTHS AND LIMITATIONS OF THIS STUDY**

- This was a representative household survey.
- All data were self-reported and some outcomes relied on recall of the past year.
- Data were collected in 2018, and there may have been changes since the COVID-19 pandemic.

## Introduction

 Most people with obesity are trying to lose weight,^1 2^ but it is unclear how they seek to achieve this. In England, national guidelines recommend that people reduce energy intake and increase physical activity, facilitated by following a support programme that helps them enact this.^3^ However, there is little evidence on the extent to which these guidelines translate into people’s weight loss efforts and actions. Despite a strong desire to lose weight among the majority of adults with obesity^4^ and a substantial weight loss industry, obesity prevalence continues to increase,^5^ suggesting further research is needed about how to connect people with effective interventions.

Randomised controlled trials show that engagement with formal weight loss programmes leads to more weight loss than self-guided weight loss attempts.^6^ General practitioners (GPs) and other primary care health professionals are well placed to refer people to effective weight control interventions and these interventions can motivate them to take action.^1 7^ A systematic review and meta-analysis of US studies found a positive association between physician weight loss advice and patient weight loss behaviours.^8^ Similarly, a 2013 cross-sectional analysis in a UK population with overweight and obesity showed health professional advice was associated with a higher proportion of patients wanting to lose weight (89% vs 61% among those not receiving advice) and attempting to lose weight (68% vs 37%).^7^ However, participants were not asked about the nature of the advice or their subsequent weight loss actions.^7^ It would guide clinical practice to understand whether a specific referral rather than general advice with no referral leads to a specific action, including attendance at a weight management service.

National clinical guidelines advise clinicians to opportunistically screen and intervene with people with a body mass index (BMI) ≥30 kg/m^2^ and refer them to a weight loss service.^9^ Evidence shows this can be effective: a two-armed trial (BWeL) found that GP advice without referral was associated with 1.04 kg weight loss, while endorsement, offer and active referral of participants to a weight management programme increased weight loss to 2.43 kg at 12 months.^6^ Evidence suggests community weight loss services are equally effective for men and women who attend^10^ but that men are less likely to be referred in the first place, which may reflect a bias among GPs or differences in patient demand, perhaps because some programmes are perceived as more suitable for women or because women make more weight loss attempts.^11^

GP records suggest that they refer only a small minority of patients to these programmes each year,^12^ despite 26% of adults in England having a BMI ≥30^5^ and the average person visiting their GP around five times per year.^13 14^ In addition, few patients are weighed regularly and weight loss advice or other weight management interventions are infrequently recorded.^15^ A potential barrier to GPs and nurses discussing weight with patients is being worried about offending patients or feeling that the topic is inappropriate or too sensitive to discuss.^16^ However, qualitative studies exploring patients’ perspectives on discussing weight in primary care settings suggest people with overweight and obesity would like to discuss their weight in consultations *more* frequently and that avoidance of the topic can give the impression to patients that they are unworthy of medical time.^17^ This evidence was supported by a trial of opportunistic brief interventions in 1882 patients, where only four patients (0.2%) found GP-delivered weight advice inappropriate and unhelpful.^6^

Using self-reported data collected as part of a nationally representative survey of adults in England, this study aimed to examine the prevalence of weight control efforts and practices, the reported frequency and impact of receipt of GP advice on weight loss attempts and perceptions of the appropriateness of health professionals delivering weight loss advice. Specific research questions were as follows.

1. Among adults in England, what proportion report that they are currently trying to lose or maintain weight and what practices do they report using?
2. Among those who have visited their GP in the last 12 months (and specifically among those with a BMI in the overweight or obesity range at the time of the survey), what proportion report having received advice on weight loss in the past year and what was the nature of this advice?
3. To what extent is reporting receipt of GP advice on weight loss in the past year associated with reporting current attempts to lose weight and different weight management practices, and do these associations differ according to the type of advice people report receiving (specific referral or prescription medication for weight loss vs general advice with no referral)?
4. Among adults in England, what proportion think it is appropriate for health professionals to deliver weight loss advice?
5. Do RQ1, RQ2 and RQ4 differ according to weight status, age, gender, ethnicity or socioeconomic position?

## Methods

### Design

Data were from the Smoking and Alcohol Toolkit Study, an ongoing monthly cross-sectional household survey representative of adults in England.^18^ The study is designed to monitor population trends in smoking and alcohol use and uses a hybrid of random location and quota sampling to select a sample of approximately 1700 adults aged ≥16 years in England each month. Data for this paper were obtained in October 2018, when additional questions on weight management were included for a single wave. Data were collected by the market research company Ipsos Mori via face-to-face computer-assisted personal interviews.

The methods have been described in detail elsewhere.^18 19^ Briefly, England was split into 165 665 output areas, each comprising approximately 300 households. These areas were then stratified according to established geodemographic characteristics and geographic regions and then randomly selected into an interviewer’s list. Interviewers travelled to their selected areas to conduct interviews with one household member aged ≥16 years. Face-to-face computer-assisted interviews were conducted until quotas based upon factors influencing the probability of being at home (ie, working status, age and gender) were fulfilled. Morning interviews were avoided to maximise participant availability.

Ethical approval for the STS was granted originally by the UCL Ethics Committee (ID 0498/001). The data are not collected by UCL and are anonymised when received by UCL. All participants provided informed consent. The study was conducted in line with the Declaration of Helsinki.

### Measures

Full details of the measures are provided in the online supplemental file.

#### Sociodemographic and anthropometric variables

Sociodemographic information included participants’ age, gender, ethnicity (ethnic minority: yes/no) and occupational social grade (ABC1 includes managerial, professional and upper supervisory occupations; C2DE includes manual routine, semiroutine, lower supervisory and long-term unemployed).

Weight and height were self-reported. We used the following criteria to identify implausible and inaccurate values, which were then treated as missing data: weight <24.9 kg (<55 pounds) or >453.6 kg (>1000 pounds) and height <111.8 cm (<44 inches) or >228.6 cm (>90 inches).^20^ For those with valid height and weight measurements, BMI was calculated as weight in kilograms divided by the square of height in metres. We further addressed implausible combinations of height and weight by excluding BMI values <12 or >70 kg/m^2^.^20^ For those with a valid BMI, weight status was categorised as underweight (<18.5 kg/m^2^), healthy weight (18.5–24.9 kg/m^2^), overweight (25–29.9 kg/m^2^) or obesity (≥30 kg/m^2^).

#### Receipt of GP advice on weight loss

Participants were asked: ‘Has your GP spoken to you about trying to lose weight in the last 12 months?’

1. Yes, he/she referred me to a weight loss group (eg, Slimming World, Weight Watchers).
2. Yes, he/she suggested that I see the practice nurse.
3. Yes, he/she referred me to the hospital for help to lose weight.
4. Yes, he/she talked to me about changing my eating and/or activity levels.
5. Yes, he/she prescribed me medication for weight loss.
6. Yes, he/she advised me to lose weight but did not suggest anything specific.
7. No, I have seen my GP in the last 12 months, but he/she did not advise me to lose weight.
8. No, I have not seen my GP in the last 12 months.

Those who responded ‘yes’ were able to select multiple responses between 1 and 6 to indicate all types of advice they received. Those who responded ‘no’ were able to select only one response option (7 or 8). We reported descriptive data on each type of advice received.

For analyses of associations with weight loss attempts and practices, we analysed receipt of any GP advice (response options 1–6 coded 1, else coded 0), receipt of a specific referral or prescription medication (response options 1, 3 and 5 coded 1, else coded 0) and receipt of general advice with no referral/medication (response options 2, 4 and 6 and not response options 1, 3 and 5 coded 1, else coded 0). Those who responded that they had not seen their GP in the last year (response 8) were excluded from prevalence estimates but were included in analyses of associations between receipt of advice and weight loss attempts and practices. We estimated the prevalence of receipt of advice among all participants who had visited their GP in the last year and specifically among those with a BMI in the overweight or obesity range at the time of the survey. While the former group included participants with a BMI in the healthy weight range, it is plausible that some of these participants may have had a higher BMI, received GP advice on weight loss and subsequently lost weight (as the question asked about receipt of advice over the past year).

#### Weight control efforts and practices

Weight control efforts were assessed among all participants with the question: ‘Which, if any, of the following apply to you?’

1. I am trying to lose weight.
2. I am trying to maintain my current weight after having lost weight.
3. I am trying to maintain my current weight.
4. I am trying to gain weight.
5. I am not trying to control my weight.

Participants could also respond ‘don’t know’ or ‘prefer not to say’. We provided descriptive data on each response option and analysed weight loss attempts as reporting ‘trying to lose weight’ versus all other responses.

Among those who reported trying to lose weight or maintain their current weight after having lost weight, current weight control practices were assessed with the question: ‘Which, if any, of the following are you currently doing to lose or maintain your weight?’ They were asked to choose all that applied from the following options:

1. I am doing more exercise and/or being more active.
2. I am eating healthily.
3. I am following a specific diet plan (eg, Atkins, blood sugar diet, 5:2).
4. I am keeping track of what I eat.
5. I am keeping track of my physical activity.
6. I am keeping track of my weight.
7. I use a weight loss service (eg, Weight Watchers) or go to see someone for help managing my weight.

#### Appropriateness of health professionals delivering weight loss advice

Participants were asked: ‘Thinking about healthcare providers asking patients about their weight at healthcare consultations, to what extent do you agree or disagree with the following statement? ‘*Weight is a personal matter and not something healthcare providers should ask about’*’ Response options were on a five-point Likert scale from strongly agree to strongly disagree. Participants could also respond ‘don’t know’ or ‘prefer not to say’.

### Statistical analysis

R V.4.2.1 was used for the analysis. The Smoking and Alcohol Toolkit Study uses raking to weight the sample to match the population of England in terms of key demographics.^18^ The following analyses used weighted data. Missing data were excluded on a per-analysis basis.

We reported the prevalence (with a 95% CI) of different weight control efforts among all participants and different weight control practices among those who reported trying to lose or maintain weight, overall and by weight status, age, gender, ethnicity and occupational social grade.

Among participants who reported visiting their GP in the last 12 months, we reported the prevalence (with 95% CI) of receipt of different types of GP advice on weight loss. We then used logistic regression to test associations of receipt of GP advice on weight loss in the last 12 months with current weight loss attempts and practices. For each outcome, we modelled the type of advice received as a three-level variable: (1) no advice (reference group), (2) receipt of general advice with no referral/medication and (3) receipt of a specific referral or prescription medication for weight loss. All models were adjusted for weight status, age, gender, ethnicity and occupational social grade.

Finally, we reported the prevalence (with 95% CI) of different perceptions of the appropriateness of health professionals delivering weight loss advice, overall and by weight status, age, gender, ethnicity and occupational social grade.

### Patient and public involvement

None.

## Results

Data were collected from 1722 participants. Weighted sample characteristics are summarised in table 1. Participants’ mean age was 47.4 years, 51.1% were women, 85.9% were white, and 44% were from less advantaged social grades. BMI estimates in the valid range (see Methods section for details) were available for 1262 participants (73.3% of the sample), of whom 4.7% (95% CI 3.6% to 6.1%) had a BMI in the underweight range, 48.1% (95% CI 45.2% to 51%) in the healthy weight range, 29.7% (95% CI 27.1% to 32.4%) in the overweight range and 17.5% (95% CI 15.4% to 19.8%) in the obesity range. Of the remainder, 378 did not report their weight and/or height (348 had missing data on weight and 177 on height) and 82 provided values deemed to be implausible (see Methods section).

**Table 1:** Sample characteristics

	% (n)*
Age (years)	
Mean (SD)	47.4 (19.2)
16–34	30.9 (532)
35–49	23.0 (396)
50–64	23.9 (411)
≥65	22.2 (382)
Gender	
Men	48.9 (841)
Women	51.1 (880)
Ethnicity	
White	85.9 (1472)
Minority group	14.1 (242)
Occupational social grade	
ABC1 (more advantaged)	56.0 (964)
C2DE (less advantaged)	44.0 (758)
Weight status	
Underweight	4.7 (59)
Healthy weight	48.1 (607)
Overweight	29.7 (375)
Obesity	17.5 (221)

All data are weighted to match the adult population in England. Sample sizes may not sum perfectly due to rounding.

There were some missing cases on gender (*n*n=1), ethnicity (*n*n=9), and weight status (*n*n=459); valid percentages are shown for ease of interpretation.

* Column percentages.

### Weight control efforts and practices

Overall, 32.8% of participants reported currently trying to lose weight, 4.7% were trying to maintain their weight after having lost weight, and 15.9% were trying to maintain their weight (table 2). The proportion trying to lose weight was substantially higher among those with overweight (45.3%) or obesity (64.7%). It was also higher among middle-aged (39.2% and 39.7% for those aged 35–49 and 50–64 years, respectively) compared with younger and older adults (29.1% and 24.1% for those aged 18–34 and ≥65 years, respectively), among women compared with men (36.6% vs 29%), and among those from more compared with less advantaged social grades (36.8% vs 27.9%) but was similar among white and ethnic minority groups (33.1% vs 32%, respectively).

**Table 2:** Prevalence of current weight control efforts among adults

	N	Weight control efforts, %* (95% CI)						
		Trying to lose weight	Trying to maintain weight after having lost weight	Trying to maintain weight	Trying to gain weight	Not trying to control weight	Don’t know	Prefer not to say
All adults	1722	32.8 (30.5 to 35.2)	4.7 (3.7 to 5.8)	15.9 (14.1 to 17.7)	3.4 (2.4 to 4.3)	35.4 (33.0 to 37.8)	2.8 (2.0 to 3.7)	4.9 (3.8 to 6.0)
Weight status								
Underweight	59	17.2 (6.4 to 28.0)	6.4 (0.7 to 12.2)	12.6 (3.4 to 21.7)	16.2 (5.4 to 27.1)	44.6 (30.8 to 58.3)	1.4 (0 to 4.3)	1.5 (0 to 4.6)
Healthy weight	607	18.6 (15.3 to 21.8)	5.4 (3.6 to 7.3)	22.9 (19.4 to 26.4)	5.1 (3.2 to 7.1)	44.0 (39.9 to 48.2)	2.8 (1.3 to 4.4)	1.1 (0.2 to 2.0)
Overweight	375	45.3 (40.1 to 50.5)	5.7 (3.3 to 8.1)	13.0 (9.5 to 16.4)	2.6 (0.8 to 4.4)	31.6 (26.6 to 36.5)	0.5 (0 to 1.2)	1.4 (0.3 to 2.4)
Obesity	221	64.7 (58.1 to 71.3)	1.6 (0.2 to 3.1)	6.6 (3.2 to 10.1)	1.3 (0 to 3.2)	21.2 (15.7 to 26.7)	3.6 (0.9 to 6.3)	0.9 (0 to 2.6)
Age (years)								
18–34	532	29.1 (24.9 to 33.4)	3.3 (1.6 to 5.0)	16.7 (13.1 to 20.2)	5.5 (3.2 to 7.7)	36.0 (31.4 to 40.6)	5.1 (3.1 to 7.0)	4.4 (2.5 to 6.3)
35–49	396	39.2 (33.8 to 44.5)	2.3 (0.7 to 3.8)	12.9 (9.2 to 16.5)	2.0 (0.4 to 3.7)	33.9 (28.8 to 39.0)	3.7 (1.5 to 6.0)	6.0 (3.6 to 8.5)
50–64	411	39.7 (34.9 to 44.6)	5.2 (2.9 to 7.5)	15.0 (11.5 to 18.5)	2.6 (0.9 to 4.2)	31.7 (27.2 to 36.3)	0.5 (0 to 1.2)	5.2 (3.0 to 7.5)
≥65	382	24.1 (20.1 to 28.2)	8.8 (6.2 to 11.5)	19.0 (15.3 to 22.7)	2.7 (1.0 to 4.4)	40.0 (35.3 to 44.7)	1.2 (0.1 to 2.2)	4.1 (2.2 to 6.0)
Gender								
Men	841	29.0 (25.7 to 32.3)	4.9 (3.4 to 6.4)	17.0 (14.3 to 19.6)	4.9 (3.3 to 6.6)	38.3 (34.8 to 41.8)	2.9 (1.7 to 4.1)	3.0 (1.7 to 4.3)
Women	880	36.6 (33.2 to 39.9)	4.6 (3.1 to 6.0)	15.0 (12.5 to 17.4)	1.9 (0.9 to 2.9)	32.7 (29.5 to 35.9)	2.6 (1.4 to 3.8)	6.7 (5.0 to 8.4)
Ethnicity								
White	1472	33.1 (30.6 to 35.6)	5.0 (3.8 to 6.1)	16.0 (14.0 to 17.9)	3.3 (2.2 to 4.3)	36.2 (33.7 to 38.8)	2.1 (1.4 to 2.9)	4.3 (3.2– to 5.4)
Minority group	242	32.0 (25.5 to 38.5)	3.5 (1.0 to 6.0)	16.3 (11.1 to 21.4)	3.9 (0.9 to 6.8)	31.0 (24.6 to 37.5)	6.6 (2.9 to 10.3)	6.7 (3.3 to 10.1)
Occupational social grade								
ABC1 (more advantaged)	964	36.8 (33.7 to 39.8)	4.5 (3.2 to 5.8)	17.3 (14.9 to 19.7)	2.7 (1.6 to 3.9)	32.2 (29.3 to 35.2)	1.9 (1.0 to 2.7)	4.6 (3.3 to 5.9)
C2DE (less advantaged)	758	27.9 (24.3 to 31.5)	5.1 (3.4 to 6.8)	14.2 (11.4 to 16.9)	4.2 (2.6 to 5.9)	39.4 (35.5 to 43.3)	4.0 (2.4 to 5.6)	5.3 (3.5 to 7.1)

All data are weighted to match the adult population in England. Sample sizes may not sum perfectly due to rounding.

* Row percentages.

Among those trying to lose or maintain weight (n=646), the most frequently reported practices were eating healthily (55.1%), doing more exercise (38.9%) and keeping track of food intake (37.4%; Table S1). Using a weight loss service or following a specific diet plan were least commonly reported (5.4% and 5.1%, respectively). The proportion who reported using a weight loss service was higher among those with a BMI in the obesity range (10.9% vs 5.7% overweight and 3.4% healthy weight), women (7.8% vs 2.4% men), those from white ethnic groups (6.1% vs 0.8% ethnic minority groups) and those from more advantaged social grades (6.4% vs 3.8% less advantaged). However, we note that the 95% CIs were wide, meaning most of these differences were uncertain.

### Receipt of GP advice on weight loss

Of the participants, 68.6% (n=1179) reported having visited their GP in the last 12 months. Among those with a BMI in the overweight or obesity range, 12.6% and 40%, respectively, recalled having received advice on weight loss (table 3). Among both groups, this was largely general advice (10.2% and 30.8%, respectively): just 2.4% of those with overweight and 9.2% of those with obesity reported receiving a referral or prescription for weight loss medication.

**Table 3:** Prevalence of receipt of GP advice on weight loss among adults who visited their GP in the last 12 months

	N	Receipt of GP advice on weight loss, % (95% CI)*			
		Received any advice†	Received referral/medication‡	Received general advice with no referral/medication§	Not advised to lose weight¶
All adults	1179	12.7 (10.8 to 14.7)	3.4 (2.3 to 4.4)	9.3 (7.6 to 11.1)	73.7 (71.1 to 76.3)
Weight status					
Underweight	36	4.6 (0 to 11.3)	0 (0 to 0)	4.6 (0 to 11.3)	88.8 (78.8 to 98.7)
Healthy weight	395	2.9 (1.4 to 4.5)	1.0 (0.1 to 1.8)	2.0 (0.7 to 3.3)	88.1 (84.8 to 91.4)
Overweight	267	12.6 (8.6 to 16.5)	2.4 (0.7 to 4.1)	10.2 (6.5 to 13.8)	78.2 (73.3 to 83.2)
Obesity	169	40.0 (32.2 to 47.7)	9.2 (4.6 to 13.7)	30.8 (23.5 to 38.2)	56.6 (48.7 to 64.4)
Age (years)					
18–34	331	8.4 (5.1 to 11.6)	3.0 (1.0 to 5.1)	5.3 (2.6 to 8.0)	77.7 (72.7 to 82.6)
35–49	261	16.0 (11.1 to 21.0)	3.7 (1.4 to 6.1)	12.3 (7.8 to 16.9)	68.3 (62.1 to 74.5)
50–64	291	14.8 (10.6 to 19.0)	4.8 (2.4 to 7.3)	10.0 (6.3 to 13.6)	72.5 (67.2 to 77.7)
≥65	296	12.7 (9.2 to 16.2)	2.1 (0.6 to 3.6)	10.6 (7.3 to 13.9)	75.1 (70.5 to 79.8)
Gender					
Men	552	11.8 (9.0 to 14.5)	2.5 (1.2 to 3.8)	9.3 (6.8 to 11.8)	75.8 (72.1 to 79.5)
Women	626	13.6 (10.8 to 16.4)	4.2 (2.6 to 5.8)	9.4 (7.0 to 11.9)	71.8 (68.1 to 75.4)
Ethnicity					
White	1007	12.4 (10.3 to 14.5)	3.2 (2.1 to 4.3)	9.3 (7.4 to 11.1)	76.0 (73.3 to 78.7)
Minority group	164	15.2 (9.2 to 21.3)	4.9 (1.5 to 8.4)	10.3 (5.1 to 15.5)	62.1 (53.9 to 70.3)
Occupational social grade					
ABC1 (more advantaged)	668	12.1 (9.7 to 14.5)	3.3 (2.0 to 4.6)	8.8 (6.7 to 10.8)	77.5 (74.4 to 80.6)
C2DE (less advantaged)	511	13.6 (10.3 to 16.9)	3.5 (1.8 to 5.2)	10.1 (7.2 to 13.1)	68.7 (64.3 to 73.2)

All data are weighted to match the adult population in England. Sample sizes may not sum perfectly due to rounding.

* Row percentages.

† Referred to weight loss programme, suggested seeing a practice nurse, referred to hospital, talked about diet and exercise, prescribed medication or non-specific advice to lose weight.

‡ Referred to weight loss programme, referred to hospital or prescribed medication.

§ Suggested see practice nurse, talked about diet and exercise or non-specific advice to lose weight.

¶ Specifically reported that they were not advised to lose weight. This is not exactly the inverse of ‘received any advice’ because some participants responded ‘don’t know’ (4.5% (95% CI 3.2%–5.8%)) or ‘prefer not to say’ (9.1% (95% CI 7.4% to 10.8%).

GPgeneral practitioner

Among participants who received GP advice on weight loss, the most common types of advice were talking about diet and exercise (44.5%) and non-specific advice to lose weight (39%); the most common type of intervention was referral to a weight loss service (14.2%; Table S2).

### Association of receipt of GP advice with weight loss attempts and practices

Among all participants, the odds of trying to lose weight were substantially higher among those who reported having received a specific referral or prescription medication for weight loss (OR_adj_ 9.25, 95% CI 2.65 to 32.3) or general advice (OR_adj_ 4.49, 95% CI 2.52 to 8) from their GP in the last 12 months (table 4).

**Table 4:** Association of receipt of GP advice on weight loss with trying to lose weight and weight control practices

	%† (95%CI)			OR_adj_(95%CI)	
	Did not receive GP advice on weight loss (1)	Received general advice (2)	Received specific referral/medication (3)	Versus (1)	Versus (1)
Among all adults‡ (n=1718)					
Trying to lose weight	28.4 (26.1 to 30.8)	77.0 (69.0 to 85.1)	85.1 (74.4 to 95.8)	4.49 (2.52 to 8.00)*	9.25 (2.65 to 32.3)*
Among adults trying to lose weight or maintain weight after having lost weight‡ (n=650)					
Doing more exercise	37.2 (32.8 to 41.6)	49.2 (38.2 to 60.3)	36.7 (19.5 to 53.9)	1.87 (1.05 to 3.33)^*^	1.13 (0.46 to 2.80)
Eating healthily	54.5 (50.0 to 59.0)	60.1 (49.3 to 71.0)	51.6 (33.7 to 69.4)	1.54 (0.86 to 2.76)	1.05 (0.44 to 2.52)
Following a specific diet plan	4.9 (2.9 to 6.9)	6.6 (1.7 to 11.6)	4.0 (0 to 9.7)	1.64 (0.46 to 5.81)	1.46 (0.20 to 10.9)
Keeping track of food intake	37.5 (33.2 to 41.9)	40.5 (29.7 to 51.2)	26.9 (11.0 to 42.8)	1.03 (0.59 to 1.80)	0.81 (0.31 to 2.12)
Keeping track of physical activity	24.9 (21.0 to 28.7)	30.7 (20.7 to 40.7)	22.9 (8.0 to 37.7)	1.35 (0.75 to 2.42)	1.18 (0.41–3.37)
Keeping track of weight	28.1 (24.1 to 32.2)	25.5 (16.2 to 34.7)	24.6 (10.1 to 39.1)	0.99 (0.52 to 1.86)	1.26 (0.49 to 3.21)
Using a commercial weight loss service	4.6 (2.8 to 6.4)	3.5 (0 to 7.5)	22.1 (6.8 to 37.3)	0.53 (0.15 to 1.83)	3.73 (1.10 to 12.6)*

All data are weighted to match the adult population in England.

* Column percentages.P<0.05.

† Whether or not they reported having visited their GP in the last .Column percentages.

‡ Whether or not they reported having visited their GP in the last 12 months.

GPgnereal practitonerOR_adj_, OR adjusted for weight status, age, gender, ethnicity and occupational social grade

Among participants who reported trying to lose weight or maintain weight after having lost weight, those who received a specific referral/medication had more than three times higher odds of reporting using a weight loss service (OR_adj_ 3.73, 95% CI 1.10 to 12.6) and those who received general advice on weight loss had higher odds of reporting doing more exercise (OR_adj_ 1.87, 95% CI 1.05 to 3.33). Other associations were uncertain.

### Appropriateness of health professionals delivering weight loss advice

Among all participants, just over half (53.5% (95% CI 51% to 55.9%)) disagreed or strongly disagreed with the statement ‘Weight is a personal matter and not something healthcare providers should ask about’; 19.3% (95% CI 17.5% to 21.4%) agreed or strongly agreed (table 5). The proportion who agreed or strongly agreed was lowest among those with a BMI in the healthy weight range (14.9% (95% CI 12.2% to 18.1%)) and highest among those with a BMI in the underweight range (28.4% (95% CI 18% to 41.8%)). The proportion who strongly agreed was higher among women compared with men (10.6% vs 6.5%) and among those from less compared with more advantaged social grades (11.0% vs 6.7%; table 5).

**Table 5:** Perceptions of the appropriateness of health professionals delivering weight loss advice among adults

	N	‘Weight is a personal matter and not something healthcare providers should ask about’, % (95% CI)						
		Strongly agree	Agree	Neither agree nor disagree	Disagree	Strongly disagree	Don’t know	Prefer not to say
All adults	1722	8.6 (7.3 to 10.1)	10.8 (9.4 to 12.3)	20.9 (19 to 23)	19.2 (17.4 to 21.3)	34.2 (31.9 to 36.6)	2.6 (1.9 to 3.5)	3.7 (2.9 to 4.8)
Weight status								
Underweight	59	7.1 (2.6 to 18.2)	21.3 (12.2 to 34.4)	24.3 (14.2 to 38.4)	11.4 (5.0 to 24.1)	35.9 (24.0 to 49.8)	0 (0 to 0)	0 (0 to 0)
Healthy weight	607	7.9 (5.9 to 10.5)	7.0 (5.2 to 9.3)	19.9 (16.7 to 23.5)	22.5 (19.2 to 26.2)	38.8 (34.9 to 43.0)	2.8 (1.6 to 4.7)	1.1 (0.5 to 2.4)
Overweight	375	8.8 (6.3 to 12.2)	11.4 (8.4 to 15.1)	19.1 (15.3 to 23.6)	17.3 (13.7 to 21.5)	40.1 (35.1 to 45.4)	1.1 (0.4 to 3)	2.3 (1.2 to 4.4)
Obesity	221	9.7 (6.3 to 14.8)	13.2 (9.3 to 18.4)	23.4 (18.0 to 29.9)	14.7 (10.5 to 20.2)	36.6 (30.2 to 43.6)	2.0 (0.8 to 4.7)	0.4 (0.1 to 2.9)
Age (years)								
18–34	532	7.6 (5.4 to 10.6)	9.7 (7.3 to 12.7)	21.9 (18.2 to 26.1)	20.7 (17.2 to 24.8)	33.3 (29 to 37.9)	3.3 (1.9 to 5.5)	3.6 (2.2 to 5.9)
35–49	396	6.9 (4.6 to 10.3)	13.3 (10.2 to 17.3)	22.6 (18.3 to 27.5)	21.4 (17.3 to 26.2)	29.4 (24.7 to 34.6)	1.5 (0.6 to 3.6)	4.9 (3.1 to 7.7)
50–64	411	10.9 (8.2 to 14.5)	8.1 (5.8 to 11.1)	18.1 (14.6 to 22.1)	16.9 (13.6 to 20.8)	40.0 (35.3 to 45)	2.7 (1.5 to 4.7)	3.3 (2 to 5.6)
≥65	382	9.2 (6.7 to 12.5)	12.6 (9.8 to 16)	21.0 (17.5 to 25.1)	17.5 (14.1 to 21.4)	34.2 (29.9 to 38.9)	2.5 (1.4 to 4.6)	3.0 (1.8 to 5.1)
Gender								
Men	841	6.5 (4.9 to 8.6)	10.1 (8.2 to 12.4)	20.3 (17.6 to 23.4)	19.0 (16.3 to 22)	38.7 (35.2 to 42.2)	2.6 (1.7 to 4)	2.8 (1.9 to 4.3)
Women	880	10.6 (8.6 to 12.9)	11.4 (9.5 to 13.7)	21.5 (18.8 to 24.5)	19.5 (16.9 to 22.4)	29.9 (26.9 to 33.1)	2.5 (1.6 to 3.9)	4.5 (3.3 to 6.2)
Ethnicity								
White	1472	8.4 (7 to 10)	10.3 (8.9 to 12)	20.7 (18.6 to 22.9)	19.8 (17.7 to 22.0)	35 (32.5 to 37.6)	2.8 (2 to 3.8)	3.1 (2.3 to 4.2)
Minority group	242	9.9 (6.3 to 15.3)	13.4 (9.4 to 18.6)	22.8 (17.4 to 29.4)	16.7 (12.2 to 22.4)	29.9 (24 to 36.7)	1.4 (0.5 to 3.8)	5.9 (3.4 to 10)
Occupational social grade								
ABC1 (more advantaged)	964	6.7 (5.3 to 8.4)	10 (8.4 to 12)	17.8 (15.5 to 20.4)	20.2 (17.8 to 22.9)	40.8 (37.7 to 44)	1.4 (0.8 to 2.3)	3 (2.1 to 4.2)
C2DE (less advantaged)	758	11 (8.8 to 13.8)	11.7 (9.4 to 14.4)	24.9 (21.6 to 28.5)	18 (15.1 to 21.2)	25.8 (22.5 to 29.4)	4.1 (2.8 to 5.9)	4.6 (3.2 to 6.6)

All data are weighted to match the adult population in England. Sample sizes may not sum perfectly due to rounding.

## Discussion

Two-thirds of people with obesity reported trying to lose weight. Receiving weight loss advice from a GP was strongly associated with a greater likelihood of trying to lose weight. However, less than half (40%) of people with obesity recalled receiving advice from their GP to lose weight in the past 12 months. Among those who recalled receiving advice, the most common action by GPs was advice to reduce food intake or increase physical activity (45%), but many people were advised to lose weight without any specific advice about how to do so (39%) and few were referred to a weight loss programme (14%). Most people considered weight loss advice from health professionals to be acceptable, though a fifth of people did not. This negative feeling was more common among people with a BMI outside of the healthy weight range (ie, underweight, overweight or obese), women, and those from more disadvantaged social grades.

Previous studies have documented positive associations between GP advice on weight loss and changes in patient weight management behaviours^7 8^ and weight loss.^21^ This study confirms and extends these findings, exploring associations between the type of advice received and different weight control practices. We found that people who recalled having received GP advice on weight loss were substantially more likely to report trying to lose weight. However, as noted in a previous study,^22^ caution is needed about attributing weight loss action to receiving GP advice, since those who reported action may have solicited advice from the GP. Of particular note, our results suggest that GPs are more likely to give general advice to change behaviour than to offer support to do so. A previous trial found that GP advice without referral led to less weight loss than endorsement, offer and active referral of participants to a weight management programme.^6^ Consistent with this, our results show that patients who report being offered a referral to a weight loss group or hospital are much more likely to report using a weight loss service or going to see someone for help managing their weight.

These findings have implications for policy and practice. Despite evidence about the effectiveness of screening and brief interventions to refer to weight management services^6^ and corresponding guidelines, this study suggests there is a lack of implementation of this evidence in practice. In our sample, more than half of people with obesity at the time of the survey who had attended primary care in the last 12 months reported that they did not receive any advice on weight loss. Of those who did receive advice, the majority reported receiving general advice to lose weight rather than referrals to services or prescription medication for weight loss, which we know lead, on average, to a greater weight loss than self-directed efforts.^6^ This study provides further evidence of the need to introduce policies to encourage implementation of the clinical guidelines, including considering measures that have been effective at changing GP behaviours in other areas, such as Quality and Outcomes Framework incentivisation or enhanced service payments.^23^ An enhanced service for weight management was introduced by National Health Service England in 2022 to support practices to engage with patients with obesity and increase rates of referral to appropriate weight management programmes.^24^ However, there may be downsides to such programmes,^25^ and they require GPs to read, accept and implement these guidelines. This survey shows that less than half of patients with obesity who saw their GP recall receiving weight-loss advice, but that receiving advice from a GP was strongly associated with trying to lose weight, suggesting GPs may be a very important influence on decision-making. Our findings suggest that unless there have been substantial changes since 2018, further training may be needed to enable obesity interventions in primary care, which will become even more pertinent as further treatment options become available (eg, the new GLP1 drugs).

Our findings show that while the general population is broadly supportive of weight interventions by healthcare providers, a substantial minority are not: 19% thought that health professionals should not ask about weight. This finding may partly reflect the wording of the question referring to ‘healthcare providers’ in general, as opposed to GPs specifically: people may be more comfortable discussing weight with a trusted GP rather than any health professional more generally. Experience from the BWeL trial shows that almost none of the patients (<1%) who received an opportunistic intervention by trained GPs found it inappropriate and unhelpful.^6^ However, people being uncomfortable with health professionals discussing weight may also be linked to weight bias and stigmatisation they have experienced in healthcare.^26 27^ Patients with obesity are sensitive to cues that the doctor is judging them negatively and may be sensitised to this from experience of flippant weight loss advice.^17^ Discussions about weight can make patients feel stigmatised and belittled, particularly when healthcare providers use language that patients find offensive.^28^ Fear of experiencing weight stigma could therefore be a reason why some participants did not want health professionals to discuss their weight. Specific GP training to initiate sensitive, effective, and non-stigmatising weight management interventions could reduce patient resistance.

The study had several limitations. First, obesity prevalence in the study was lower than figures from the Health Survey for England (17.5% (95% CI 15.4 to 19.8%) vs 25.9% (95% CI 24.5% to 27.4%)).^5^ A quarter of participants in the current study did not report their weight and it seems plausible that people with a higher BMI were more likely to decline to report their weight (eg, as a result of shame or perceived stigma). Second, the study was partly reliant on the accuracy of recall of GP appointments in the last 12 months—including both whether they had seen their GP in this period and interactions about weight within these appointments. Only 69% of participants reported seeing their GP in the past year, when the true prevalence, based on primary care records, is likely closer to 80%.^29^ This may have led to an underestimation of receipt of advice. Recall of receipt of advice on weight loss may be subject to bias: it is possible that people who were already taking action to reduce their weight may have been more likely to discuss it with a GP or that people taking action to lose weight recall GP advice in a way that those who are not may tend to forget. Thirdly, there may be some misclassification: BMI data were based on current self-reported weight at the time of the survey, but the medical advice could have been received at any time during the past 12 months. Therefore, it is possible that some participants may have received advice while having overweight or obesity and subsequently lost weight, meaning their weight status had changed by the time they were surveyed. A few participants with an underweight BMI reported having received GP advice on weight loss; it is likely this was not advice to lose weight but may have been advice on how to reverse or prevent further weight loss. It is also possible that GPs provided advice incorrectly due to misclassification or participants mistakenly recalled having received such advice. Fourth, the data were collected in 2018 and weight control efforts and receipt of GP advice may have changed since then, particularly in the context of the COVID-19 pandemic, given that excess weight was linked to an increased risk of adverse COVID outcomes.^30^ There was also a shift to online and telephone consultations during the pandemic, so there is a need to assess whether this affected the delivery of advice on weight loss or its impact on patient weight control behaviour. Finally, we could not assess whether there were other factors and motivations that influenced the likelihood of uptake and actions associated with the GP advice/referral because the survey did not include other motivations to lose weight nor whether the consultations were prescheduled reviews of obesity.

In conclusion, most people with obesity reported trying to lose weight but less than half reported having received advice on weight loss from their GP in the past 12 months. Approximately one in five thought health professionals discussing weight was inappropriate. Those who recalled having received GP advice on weight loss were substantially more likely to report taking action to lose weight. Most patients who reported receiving advice recalled being encouraged to self-manage their weight; few were referred to community weight loss programmes.

## supplementary material

10.1136/bmjopen-2024-086764 online supplemental file 1

## Data Availability

Data are available upon reasonable request.
