# Supplementary material for: Weight control efforts and practices and health professional advice: a cross-sectional national survey in England
Source: BMJ Open. 2024 Nov 11;14(11):e086764. doi: 10.1136/bmjopen-2024-086764 (PMC11555096; doi:10.1136/bmjopen-2024-086764)
Supplement: online supplemental file 1 [file bmjopen-14-11-s001.docx]

**Questionnaire items**

ASK ALL AGED 16+ IN ENGLAND

**QIMW143_01.** What is your weight? Please give this as your weight when you are not wearing shoes.

Please select how you would prefer to answer this question below.

**SINGLE CODE**

1. Stones (st) and pounds (lbs)

2. Pounds (lbs)

3. Kilograms (kg)

3. Don’t know

4. Prefer not to say

ASK ALL WHO ARE WILLING TO PROVIDE THEIR WEIGHT (QIMW146_01=1-3)

**QIMW143_02.** What is your weight? Please give this as your weight when you are not wearing shoes.

If you are unsure, please give your best estimate

**NUMERIC**

1. [INSERT NUMERIC ALLOW RANGE 0-50] st [INSERT NUMERIC ALLOW RANGE 0-13 IF 1 AT QIMW143_01.] lbs [ONLY SHOW IF 1 AT QIMW143_01]

2. **[**INSERT NUMERIC ALLOW RANGE 0-700 IF 2 AT QIMW143_01] lbs [ONLY SHOW IF 2 AT QIMW143_01]

3. [INSERT NUMERIC ALLOW RANGE 0-400] kg [ONLY SHOW IF 3 AT QIMW143_01]

4. Don’t know

5. Prefer not to say

ASK ALL AGED 16+ IN ENGLAND

**QIMW143_03.** What is your height? Please give this as your height when you are not wearing shoes.

Please select how you would prefer to answer this question below.

**SINGLE CODE**

1. Feet (ft) and inches

2. Centimetres (cm)

3. Don’t know

4. Prefer not to say

ASK ALL AGED 16+ IN ENGLAND WHO ARE WILLING TO GIVE THEIR HEIGHT (QIMW143_03 = 1 OR 2)

**QIMW143_04.** What is your height? Please give this as your height when you are not wearing shoes.

If you are unsure, please give your best estimate

**NUMERIC**

1. [INSERT NUMERIC ALLOW RANGE 4-7] ft [INSERT NUMERIC ALLOW RANGE 0-11] inches [ONLY SHOW IF 1 AT QIMW143_03]

2. [INSERT NUMERIC ALLOW RANGE 120-210] cm [ONLY SHOW IF 2 AT QIMW143_03]

3. Don’t know

4. Prefer not to say

ASK ALL AGED 16+ IN ENGLAND

**QIMW143_05.** Which, if any, of the following apply to you?.

**SINGLE CODE**

1. I am trying to lose weight
2. I am trying to maintain my current weight after having lost weight
3. I am trying to maintain my current weight
4. I am trying to gain weight
5. I am not trying to control my weight
6. Don’t know
7. Prefer not to say

ASK ALL AGED 16+ IN ENGLAND

**QIMW143_06.** Has your GP spoken to you about trying to lose weight in the last 12 months? Please choose all that apply.

**MULTI CODE 1-6, SINGLE CODE 7-10, RANDOMISE 1-6, FIX 7-10 TO BOTTOM**

1. Yes, he/she referred me to a weight loss group (e.g. Slimming World, Weight Watchers)
2. Yes, he/she suggested that I see the practice nurse
3. Yes, he/she referred me to the hospital for help to lose weight
4. Yes, he/she talked to me about changing my eating and/or activity levels
5. Yes, he/she prescribed me medication for weight loss
6. Yes, he/she advised me to lose weight but did not suggest anything specific
7. No, I have seen my GP in the last 12 months, but he/she did not advise me to lose weight
8. No, I have not seen my GP in the last 12 months
9. Don’t know
10. Prefer not to say

ASK ALL AGED 16+ IN ENGLAND WHO ARE TRYING TO LOSE WEIGHT OR MAINTAIN THEIR WEIGHT LOSS AFTER LOSING WEIGHT (QIMW142_06 = 1 OR 2)

**QIMW143_07.** Which, if any, of the following are you currently doing to lose or maintain your weight? Please choose all that apply.

**MULTI CODE 1-7, SINGLE CODE 8-9, RANDOMISE 1-7, FIX 4-6 TOGETHER AND RANDOMISE WITHIN**

1. I am doing more exercise and/or being more active
2. I am eating healthily
3. I am following a specific diet plan (e.g. Atkins, Blood sugar diet, 5:2)
4. I am keeping track of what I eat
5. I am keeping track of my physical activity
6. I am keeping track of my weight
7. I use a weight loss service (e.g. Weight Watchers) or go to see someone for help managing my weight
8. Don’t know
9. Prefer not to say

ASK ALL AGED 16+ IN ENGLAND

**QIMW143_08.** Thinking about health care providers asking patients about their weight at health care consultations, to what extent do you agree or disagree with the following statement?:

"Weight is a personal matter and not something health care providers should ask about"

**SINGLE CODE, FORWARD/REVERSE 1-5**

1. Strongly agree
2. Slightly agree
3. Neither agree nor disagree
4. Slightly disagree
5. Strongly disagree
6. Don’t know
7. Prefer not to say

##### **Table S1.** Prevalence of current weight control practices among adults trying to lose weight or maintain their current weight after having lost weight

|  |  |  | **Weight control practices^1^, %^2^ [95%CI]** | | | | | |
| --- | --- | --- | --- | --- | --- | --- | --- | --- |
|  | ***n*** | **Doing more exercise** | **Eating healthily** | **Following a specific diet plan** | **Keeping track of food intake** | **Keeping track of physical activity** | **Keeping track of weight** | **Using a weight loss service** |
|  |  |  |  |  |  |  |  |  |
| All adults | 646 | 38.9 [34.9–42.8] | 55.1 [51.1–59.1] | 5.1 [3.3–6.8] | 37.4 [33.5–41.3] | 25.6 [22.1–29.1] | 27.6 [24.0–31.2] | 5.4 [3.6–7.2] |
|  |  |  |  |  |  |  |  |  |
| Weight status |  |  |  |  |  |  |  |  |
| Underweight | 14 | 27.6 [0.6–54.6] | 60.5 [30.1–90.9] | 8.2 [0–25.1] | 63.5 [35.6–91.4] | 56.2 [25.8–86.6] | 38.1 [8.4–67.7] | 8.2 [0–25.1] |
| Healthy weight | 146 | 41.1 [32.7–49.4] | 63.8 [55.6–72.0] | 9.2 [4.0–14.5] | 33.6 [25.6–41.5] | 23.2 [16.2–30.1] | 28.0 [20.5–35.4] | 3.4 [0.4–6.4] |
| Overweight | 191 | 41.2 [34.0–48.4] | 58.2 [51.1–65.4] | 4.0 [1.3–6.6] | 40.8 [33.7–48.0] | 26.6 [20.1–33.0] | 28.9 [22.4–35.4] | 5.7 [2.4–8.9] |
| Obesity | 147 | 43.1 [34.6–51.7] | 51.6 [43.0–60.2] | 5.4 [1.6–9.1] | 39.3 [30.9–47.7] | 31.5 [23.4–39.7] | 30.2 [22.3–38.1] | 10.9 [5.7–16.1] |
|  |  |  |  |  |  |  |  |  |
| Age (years) |  |  |  |  |  |  |  |  |
| 18-34 | 173 | 47.8 [39.6–56.1] | 53.6 [45.4–61.8] | 5.3 [1.3–9.3] | 41.3 [33.2–49.4] | 30.6 [23.1–38.0] | 28.6 [21.0–36.1] | 6.4 [2.6–10.1] |
| 35-49 | 162 | 44.5 [36.0–53.0] | 51.8 [43.2–60.3] | 7.1 [3.0–11.3] | 28.8 [21.1–36.5] | 24.9 [17.7–32.2] | 24.6 [17.4–31.8] | 4.2 [0.7–7.7] |
| 50-64 | 185 | 35.1 [28.0–42.1] | 56.7 [49.3–64.1] | 3.1 [0.6–5.7] | 36.9 [29.7–44.1] | 22.6 [16.4–28.9] | 23.6 [17.3–29.9] | 6.2 [2.9–9.6] |
| ≥65 | 126 | 24.9 [17.8–32.0] | 59.2 [51.2–67.2] | 4.9 [1.5–8.4] | 43.8 [35.6–51.9] | 24.0 [17.0–30.9] | 35.9 [28.0–43.7] | 4.3 [0.8–7.8] |
|  |  |  |  |  |  |  |  |  |
| Gender |  |  |  |  |  |  |  |  |
| Men | 284 | 44.2 [38.0–50.3] | 56.4 [50.3–62.6] | 2.9 [1.0–4.9] | 38.1 [32.2–44.1] | 31.7 [25.9–37.4] | 35.3 [29.5–41.1] | 2.4 [0.6–4.2] |
| Women | 361 | 34.7 [29.6–39.8] | 54.1 [48.7–59.4] | 6.7 [4.0–9.5] | 36.8 [31.6–42.0] | 20.8 [16.6–25.0] | 21.5 [17.0–25.9] | 7.8 [5.0–10.6] |
|  |  |  |  |  |  |  |  |  |
| Ethnicity |  |  |  |  |  |  |  |  |
| White | 559 | 38.1 [33.9–42.3] | 58.2 [53.9–62.5] | 5.4 [3.4–7.4] | 38.9 [34.7–43.1] | 25.7 [22.0–29.5] | 28.6 [24.7–32.4] | 6.1 [4.1–8.1] |
| Minority group | 86 | 44.3 [32.6–56.0] | 35.9 [24.7–47.2] | 2.9 [0–6.3] | 28.1 [17.9–38.4] | 25.1 [15.0–35.3] | 21.7 [11.9–31.4] | 0.8 [0–2.5] |
|  |  |  |  |  |  |  |  |  |
| Occupational social grade |  |  |  |  |  |  |  |  |
| ABC1 | 397 | 42.1 [37.2–47.0] | 56.9 [52.0–61.7] | 5.5 [3.3–7.7] | 38.5 [33.7–43.2] | 31.3 [26.7–35.9] | 32.3 [27.6–36.9] | 6.4 [4.0–8.8] |
| C2DE | 249 | 33.7 [27.0–40.4] | 52.3 [45.3–59.4] | 4.5 [1.5–7.4] | 35.7 [29.0–42.3] | 16.5 [11.3–21.7] | 20.1 [14.6–25.7] | 3.8 [1.3–6.3] |
|  |  |  |  |  |  |  |  |  |

CI, confidence interval. Social grades ABC1 = more advantaged, C2DE = less advantaged.

All data are weighted to match the adult population in England. Sample sizes may not sum perfectly due to rounding.

^1^ Participants were asked to report all that apply.

^2^ Row percentages.

##### **Table S2**. Prevalence of receipt of specific types of GP advice on weight loss among adults who received any GP advice on weight loss in the last 12 months

|  |  | **Receipt of GP advice on weight loss, %^1^ [95%CI]** | | | | | |
| --- | --- | --- | --- | --- | --- | --- | --- |
|  | ***n*** | **Referred to weight loss programme** | **Suggested see practice nurse** | **Referred to hospital** | **Talked about diet and exercise** | **Prescribed medication** | **Non-specific advice to lose weight** |
|  |  |  |  |  |  |  |  |
| All adults | 150 | 14.2 [8.4–20.1] | 9.0 [4.3–13.7] | 2.6 [0.3–4.8] | 44.5 [36.2–52.9] | 3.3 [0.6–5.9] | 39.0 [30.8–47.2] |
|  |  |  |  |  |  |  |  |
| Weight status^2^ |  |  |  |  |  |  |  |
| Healthy weight | 12 | 6.2 [0–19.4] | 14.9 [0–35.8] | 5.3 [0–16.7] | 30.2 [2.7–57.8] | 6.4 [0–19.9] | 36.9 [8.4–65.4] |
| Overweight | 34 | 4.9 [0–11.9] | 5.1 [0–12.6] | 2.1 [0–6.3] | 43.0 [25.9–60.0] | 6.9 [0–14.9] | 44.3 [27.3–61.4] |
| Obesity | 68 | 14.2 [5.2–23.1] | 9.1 [1.9–16.3] | 2.3 [0–5.6] | 49.3 [36.4–62.2] | 2.7 [0–6.5] | 37.6 [25.1–50.1] |
|  |  |  |  |  |  |  |  |
| Age (years) |  |  |  |  |  |  |  |
| 18-34 | 28 | 24.1 [5.9–42.2] | 12.3 [0–26.4] | 0 [0–0] | 51.1 [29.7–72.6] | 0 [0–0] | 44.3 [22.9–65.7] |
| 35-49 | 42 | 14.9 [3.1–26.7] | 4.7 [0–11.5] | 1.7 [0–5.1] | 39.3 [22.3–56.2] | 1.8 [0–5.5] | 39.7 [22.6–56.9] |
| 50-64 | 43 | 15.6 [4.2–27.0] | 13.5 [3.0–24.0] | 3.8 [0–9.1] | 44.3 [28.4–60.2] | 4.3 [0–10.3] | 30.9 [16.5–45.4] |
| ≥65 | 38 | 4.8 [0–11.4] | 6.3 [0–13.4] | 4.0 [0–9.7] | 45.7 [30.7–60.7] | 6.2 [0–13.2] | 43.6 [28.5–58.6] |
|  |  |  |  |  |  |  |  |
| Gender |  |  |  |  |  |  |  |
| Men | 65 | 10.7 [2.8–18.5] | 10.2 [2.7–17.6] | 2.3 [0–5.6] | 49.8 [37.1–62.5] | 3.7 [0–7.9] | 35.1 [23–47.2] |
| Women | 85 | 17 [8.5–25.5] | 8.1 [2.0–14.2] | 2.7 [0–5.9] | 40.5 [29.4–51.6] | 3.0 [0–6.4] | 42.0 [30.7–53.2] |
|  |  |  |  |  |  |  |  |
| Ethnicity |  |  |  |  |  |  |  |
| White | 125 | 12.9 [6.8–19.1] | 9.0 [3.9–14.1] | 2.5 [0–5.0] | 45.1 [36.0–54.2] | 3.9 [0.8–7.1] | 40.4 [31.5–49.3] |
| Minority group | 25 | 20.8 [2.8–38.8] | 8.8 [0–21.2] | 2.8 [0–8.6] | 41.5 [19.8–63.1] | 0 [0–0] | 32.1 [9.7–54.5] |
|  |  |  |  |  |  |  |  |
| Occupational social grade |  |  |  |  |  |  |  |
| ABC1 | 81 | 15.4 [7.4–23.4] | 7.2 [1.9–12.4] | 2.9 [0–6.2] | 46.0 [35.3–56.8] | 3.3 [0–7.0] | 35.6 [25.3–45.8] |
| C2DE | 70 | 12.9 [4.2–21.7] | 11.1 [3.0–19.3] | 2.2 [0–5.3] | 42.8 [29.6–56.0] | 3.3 [0–7.1] | 43.0 [29.7–56.3] |
|  |  |  |  |  |  |  |  |

CI, confidence interval. Social grades ABC1 = more advantaged, C2DE = less advantaged.

All data are weighted to match the adult population in England. Sample sizes may not sum perfectly due to rounding.

^1^ Row percentages.

^2^ Data are not reported for those with a BMI in the underweight range as only two participants in this group reported having received any GP advice on weight loss.
