# Supplementary material for: Weight control efforts and practices and health professional advice: a cross-sectional national survey in England
Source: BMJ Open. 2024 Nov 11;14(11):e086764. doi: 10.1136/bmjopen-2024-086764 (PMC11555096; doi:10.1136/bmjopen-2024-086764)
Supplement: Supplementary file 2 [file bmjopen-2024-086764.reviewer_comments.pdf]

PEER REVIEW HISTORY

BMJ Open publishes all reviews undertaken for accepted manuscripts. Reviewers are asked to complete a checklist review form and are provided with free text boxes to elaborate on their assessment. These free text comments are reproduced below.

ARTICLE DETAILS

Title (Provisional)

Weight control behaviours and health professional recommendations in England: a cross-sectional national survey

Authors

Warr, William; Brown, Jamie; Hartmann-Boyce, Jamie; Jebb, Susan A; Tudor, Kate; Aveyard, Paul

---

VERSION 1 - REVIEW

**Reviewer**	**1**
**Name**	**Sturgiss, Elizabeth**
**Affiliation**	**Monash University, Department of General Practice**
**Date**	**31-Aug-2022**
**COI**	**I am a primary care researcher in the area of obesity management. I have no industry connections.**

Thank you for the opportunity to review this nationally representative survey of adults in England investigating reported GP referral/advice for those with obesity.

Abstract

- please clarify the objective by combining sentence two and three.

- it is unclear in the abstract if only those with a high weight were included? Or is it all respondents?

- "Participants were almost evenly divided over whether GPs should ask about weight." - please re-word as the question in the survey was about all health care providers, not GPs.

- " A minority (15%) of all people reported receiving weight loss advice from a GP in the past year but nearly half (42%) of those with obesity received advice." - I am unclear why you were interested in GPs giving weight loss advice to people of healthy/under weight? The first part of the sentence seems to reflect poorly on GPs, but it is the total population sampled, not only those reporting a higher BMI, so it doesn't make clinical sense to me?

- in the results of the abstract, please make it clear which ones a the total sample and which are people reporting higher body weight.

Strengths and Limitations are clear

Throughout - please soften the language to "patient recall", or "patient remembered being told by GP". Many of the sentences in the results and discussion imply that there has been a more robust analysis of what GPs did than patient recall.

Introduction

- National guidelines - please clarify for the global reader

- page 3, HP? is this GP?

- can you please describe how accessible weight loss programs are in England?

- It would be helpful to have the aim/objective directly stated before the methods (at the end of the introduction)

Methods

- The data is from 2018, a lot has changed in the world since then. Are there limitations with data from 2018 about GP activity considering the changes to GP in the UK since then? (e.g. telehealth, availability of weight management programs)

Results

- did any people with BMI <25 report trying to lose weight? also for those 25-30? (currently only <30 is reported).

- only 59% reported seeing a GP, this seems quite low compared to national standards for GP access? (GP Patient Survey, UK) I see that this is mentioned in the limitations, but no comment is made on the influence of this finding on the results.

- "Of the total population, 1020 (59%) reported having seen their GP within the last 12 months. Of those, 151/1020 (15%) reported receiving weight loss advice." I'm unsure why the denominator is the total sample? Why would you expect the GP to give weight loss advice to those of healthy or under-weight?

- "Of those who saw their GP, most were either advised to change their eating/activity habits (6%; 57/1020, 6%) or advised to lose weight but without advice on how to achieve this (4%; 45/1020) and reported being referred to a community weight loss service (2%; 21/1020)" Again I do not understand why the denominator is the total sample.

The next section that breaks the advice/referrals in weight categories makes much more clinical sense.

Suggest just keeping the part that is broken into the weight categories.

- "For people with a healthy weight, 70% (7/10) of people who received advice were taking action to lose weight, vs 15% (89/578) who did not receive advice. A logistic regression

showed receiving weight loss advice was associated with trying to lose weight (unadjusted OR 13.6, 95% CI 3.2-46.2 p<0.001; adjusted OR 12.3, 95% CI 3.1-49.0, p<0.001)" - Is this a worrying finding? Does it point to weight stigma/disorder eating? It is not mentioned in the discussion.

- Question 5 in the results should be re-worded, the survey asked about health care providers, not GPs specifically.

Discussion

-" less than half (42%) of people with obesity reported receiving advice

from their GP to lose weight." - please include that this was within the last 12 months only

- again in the last sentence, it was health care providers, not GPs asked about in the survey

- a recent meta-analysis from Madigan et al in the BMJ seems relevant for the section on comparison to other literature https://www.bmj.com/content/377/bmj-2021-069719

- weight stigma is hinted at, but not explicitly mentioned: " it seems plausible that people with a higher BMI were more likely to decline to report their weight." Consider adding weight stigma/shame here; this is especially true as the survey is done face-to-face with an interviewer

- it is not very clear why smoking is mentioned in the comparisons with other literature? Perhaps research on implementation of obesity management in primary care would be more relevant

- the discussion suggests QOF incentivisation without exploring the downsides of such programs, especially for GPs working in low SES areas (where obesity prevalence is also higher). See this recent review https://www.ncbi.nlm.nih.gov/pmc/articles/PMC7880106/

- The paragraph beginning - "Our findings show that whilst the general population are supportive of weight interventions from GPs, a substantial minority are not, particularly among people with excess weight" - again needs to be changed from GPs, to all health care providers. This may also change this paragraph as the survey asked about ALL health care providers, and your quesitons only asked about advice/referral from a GP. The survey respondents may have been imaging very diverse settings for all health care providers (e.g. emergency dept, pharmacy setting, etc etc).

This is another section where weight stigma/shame would seem to be very relevant.

**Reviewer**	**2**
**Name**	**Santos, Inês**
**Affiliation**	
**Date**	**14-Dec-2022**

**COI**	**None**

This is an interesting, organized, and well-written paper on weight control intentions and practices among adults in England and health professionals advice on this regard. Although the study objective needs to be refined and some references need to be updated, the methods are appropriate, and the discussion adequately represents the main results. The findings are not particularly novel, since weight loss attempts and the impact of health professional recommendations on these attempts have been previously explored in England (Piernas C et al, Int J Obes 2016; Jackson et al, BMJ Open 2013), but there is a need for more recent data. The authors should make this clearer in the introduction and strengths section. Also, I have a few suggestions that may be of use to further enhance the clarity and quality of the overall manuscript.

Title: I believe "Weight loss attempts and practices, and health professional advice in England: a cross-sectional national survey" is more reflective of the content of the manuscript.

Introduction:

(Page 2) The first paragraph needs to be reformulated/updated. There is a systematic review on weight loss attempts published in 2017 that includes more recent data than reference #1 and reports the strategies used around the world to try to lose weight. I believe reference #2 is not an obesity guideline, but a NHS weight loss plan/app. Proper obesity guidelines should be mentioned instead.

(Page 3, lines 27-31) The literature supports that women report more weight loss attempts than men – this should be reflected here.

(Page 3, last paragraph) The stated purpose does not accord with the work that is presented – the authors are not testing impact of the health professional advice, but, instead, associations between advice and attempts. This need to be clarified here.

Methods:

(Page 4, study design and sample) No need to repeat that data was collected in one month in October 2018. Authors should correct a typo: STS instead of SATS.

Results:

(Pages 6-15) Some relative frequencies in the text don't match with relative frequencies in tables/figures (e.g., in Table 1 % of men and women; in Fig 3 % of eating healthily) – Authors should cross-check the results section. In the text, the authors are mostly using people-first language regarding obesity, but not in Tables and Figures – this should be corrected (people with overweight/obesity instead of overweight/obese people).

(Page 6, line 56) State the BMI for obesity; also, BMI should be first mentioned as body mass index.

(Page 8, first paragraph) It seems that the authors are reporting comparisons between BMI categories instead of associations – this should be reframed; line 19: Please correct typo: “maintain weight after having lost weight” instead of “maintain weight having lost weight”.

Figures 1 and 2 can be merged in a bar chart instead of having two pie charts – it will facilitate the reading having bars for the entire sample and bars for the ones with a BMI above 30.

(Page 10, lines 32-38) If there was no difference in the frequency of advice between men and women, how can one be more likely to be referred than the other? The authors can state a tendency, but only that. (Lines 50-53) Again, by how the results are stated, it seems between group comparisons and not odds ratios…

Discussion:

Since weight loss attempts and the impact of health professional recommendations on these attempts have been previously explored in England and the novelty of this paper is having more recent data, the authors should reflect on how this may have changed since the last survey, although data may not have been collected in the same way.

Strengths and limitations should be moved to the end of the discussion section.

(Page 17) State all limitations together in the strengths and limitations section and not some of them within the comparisons with other literature section.

(Pages 17-18, lines 53-4) If it wasn’t statistically different the authors can’t say it was greater…

The entire text would benefit from attention to detail – typos, spaces between lines and words, parenthesis missing etc.

Dear Editor

We apologise for the delay in responding to your invitation to submit a revised version of this paper.

The work was previously led by someone who no longer had capacity to take this paper forwards. As a colleague of co-authors on the paper with an interest in the topic, I agreed to take over the revisions. On reviewing the original submission in light of the feedback received, I and my co-authors took the opportunity to substantially improve the manuscript by reanalysing the data and changing the presentation of the results. We have rerun all of the analyses, rewritten the methods and results sections, and updated the rest of the paper to include up-to-date references and reflect the updated results. This has resulted in considerable changes from the version we originally submitted, which is why we have not attached a version with changes tracked.

Below we respond to the reviewer's feedback on our original submission with details of the changes we have made to accommodate their suggestions.

We hope you find the revised version much improved.

Best wishes
Dr Sarah Jackson

Reviewer: 1

Thank you for the opportunity to review this nationally representative survey of adults in England investigating reported GP referral/advice for those with obesity.

Abstract
- please clarify the objective by combining sentence two and three.
Response: We now state the objective as 'This study examined the prevalence of weight control efforts and practices, the reported frequency and impact of receipt of GP advice on weight-loss attempts, and perceptions of the appropriateness of health professionals delivering weight-loss advice.'

- it is unclear in the abstract if only those with a high weight were included? Or is it all respondents?
Response: We specify that participants were '1,722 adults (≥16y) surveyed in October 2018 (mean[SD] age = 47.4[19.2], 51.1% women).' We refer to different weight statuses in the results section 'People with a BMI outside of the healthy-weight range (underweight/overweight/obesity), women, and those from more disadvantaged social grades were less likely to find it acceptable.'

- "Participants were almost evenly divided over whether GPs should ask about weight." - please re-word as the question in the survey was about all health care providers, not GPs.
Response: We now say: 'Views on whether health professionals should deliver weight-loss advice were mixed, with a substantial minority (19.4% [17.5-21.4%]) finding it unacceptable.'

- " A minority (15%) of all people reported receiving weight loss advice from a GP in the past year but nearly half (42%) of those with obesity received advice." - I am unclear why you were interested in GPs giving weight loss advice to people of healthy/under weight? The first part of the sentence seems to reflect poorly on GPs, but it is the total population sampled, not only those reporting a higher BMI, so it doesn't make clinical sense to me?
Response: We now report data on receipt of GP advice among participants with obesity: 'Of people with obesity who visited their GP in the past year, 40.0%[32.2-47.7%] recalled receiving any advice on weight loss: 30.8%[23.5-38.2%] general advice and 9.2%[4.6-13.7%] a referral to a weight-loss service or prescription medication for weight loss.'

- in the results of the abstract, please make it clear which ones a the total sample and which are people reporting higher body weight.
Response: We have done this to the best of our ability within the confines of the word limit, by specifying where results applied to subgroups of participants (e.g., 'Two-thirds (64.7% [95%CI=58.1-71.3%]) of people with obesity reported trying to lose weight. Of people with obesity who visited their GP in the past year, 40.0%[32.2-47.7%] recalled receiving any advice on weight loss…'

Strengths and Limitations are clear

Throughout - please soften the language to "patient recall", or "patient remembered being told by GP". Many of the sentences in the results and discussion imply that there has been a more robust analysis of what GPs did than patient recall.
Response: We have done this throughout the manuscript.

Introduction
- National guidelines - please clarify for the global reader
Response: We have edited to 'National clinical guidelines'.

- page 3, HP? is this GP?
Response: We have corrected this typo.

- It would be helpful to have the aim/objective directly stated before the methods (at the end of the introduction)
Response: We now state the aim and research questions at the end of the introduction.

Methods
- The data is from 2018, a lot has changed in the world since then. Are there limitations with data from 2018 about GP activity considering the changes to GP in the UK since then? (e.g. telehealth, availability of weight management programs)
Response: We now address this in the discussion of the study's limitations:
'Fourthly, the data were collected in 2018 and weight control efforts and receipt of GP advice may have changed since then, particularly in the context of the COVID-19 pandemic given excess weight was linked to increased risk of adverse COVID outcomes.[25] There was also a shift to online and telephone consultations during the pandemic, so there is a need to assess whether this affected the delivery of advice on weight loss or its impact on patient weight control behaviour.'

Results

- did any people with BMI <25 report trying to lose weight? also for those 25-30? (currently only <30 is reported).
Response: We now report data on weight control efforts by weight status (see Table 2).

- only 59% reported seeing a GP, this seems quite low compared to national standards for GP access? (GP Patient Survey, UK) I see that this is mentioned in the limitations, but no comment is made on the influence of this finding on the results.
Response: Our reanalysis shows the weighted proportion who reported seeing a GP was 68.6%. We now comment on the potential influence on the results in the discussion: 'Only 69% of participants reported seeing their GP in the past year when the true prevalence, based on primary care records, is likely closer to 80%.[24] This may have led to underestimation of receipt of advice.'

- "Of the total population, 1020 (59%) reported having seen their GP within the last 12 months. Of those, 151/1020 (15%) reported receiving weight loss advice." I'm unsure why the denominator is the total sample? Why would you expect the GP to give weight loss advice to those of healthy or under-weight?
Response: We now report the proportion receiving weight loss advice, overall and by weight status, because it is possible that some participants may have received weight loss advice up to a year before the survey and subsequently lost weight, resulting in a change in weight status.

- "Of those who saw their GP, most were either advised to change their eating/activity habits (6%; 57/1020, 6%) or advised to lose weight but without advice on how to achieve this (4%; 45/1020) and reported being referred to a community weight loss service (2%; 21/1020)" Again I do not understand why the denominator is the total sample.
The next section that breaks the advice/referrals in weight categories makes much more clinical sense.
Suggest just keeping the part that is broken into the weight categories.
Response: We provide data by weight status and sociodemographic characteristics in Table 3 to present all the available data for interested readers, but focus on the results for those with a BMI in the overweight/obesity range in the results text.

- "For people with a healthy weight, 70% (7/10) of people who received advice were taking action to lose weight, vs 15% (89/578) who did not receive advice. A logistic regression showed receiving weight loss advice was associated with trying to lose weight (unadjusted OR 13.6, 95% CI 3.2-46.2 p<0.001; adjusted OR 12.3, 95% CI 3.1-49.0, p<0.001)" - Is this a worrying finding? Does it point to weight stigma/disorder eating? It is not mentioned in the discussion.
Response: We no longer provide data on associations between receipt of advice and trying to lose weight among participants with a healthy BMI due to small numbers. Instead, we focus on the overall association and associations with different weight control practices among adults trying to lose weight or maintain weight after having lost weight.

- Question 5 in the results should be re-worded, the survey asked about health care providers, not GPs specifically.
Response: We have been careful to use the correct wording throughout the revised manuscript.

Discussion

-" less than half (42%) of people with obesity reported receiving advice from their GP to lose weight." - please include that this was within the last 12 months only
Response: We now specify the time period throughout the manuscript to ensure this is clear.

- again in the last sentence, it was health care providers, not GPs asked about in the survey
Response: See response above.

- a recent meta-analysis from Madigan et al in the BMJ seems relevant for the section on comparison to other literature https://www.bmj.com/content/377/bmj-2021-069719
Response: We now cite this study in the discussion:
'Previous studies have documented positive associations between GP advice on weight loss and changes in patient weight management behaviours[8,9] and weight loss.[21]'

- weight stigma is hinted at, but not explicitly mentioned: " it seems plausible that people with a higher BMI were more likely to decline to report their weight." Consider adding weight stigma/shame here; this is especially true as the survey is done face-to-face with an interviewer
Response: We have edited to 'it seems plausible that people with a higher BMI were more likely to decline to report their weight (e.g., as a result of shame or perceived stigma).'

- it is not very clear why smoking is mentioned in the comparisons with other literature? Perhaps research on implementation of obesity management in primary care would be more relevant
Response: We no longer mention smoking in the discussion.

- the discussion suggests QOF incentivisation without exploring the downsides of such programs, especially for GPs working in low SES areas (where obesity prevalence is also higher). See this recent review https://www.ncbi.nlm.nih.gov/pmc/articles/PMC7880106/
Response: We now acknowledge this in the discussion, citing this review:
'This study provides further evidence of the need to introduce policies to encourage implementation of the clinical guidelines, including considering measures that have been effective at changing GP behaviours in other areas, such as QOF incentivisation or Enhanced Service Payments.[23] An Enhanced Service for weight management was introduced by NHS England in 2022 to support practices to engage with patients with obesity and increase rates of referral to appropriate weight management programmes.[24] However, there may be downsides of such programmes,[25] and they require GPs to read, accept, and implement these guidelines.'

- The paragraph beginning - "Our findings show that whilst the general population are supportive of weight interventions from GPs, a substantial minority are not, particularly among people with excess weight" - again needs to be changed from GPs, to all health care providers. This may also change this paragraph as the survey asked about ALL health care providers, and your questions only asked about advice/referral from a GP. The survey respondents may have been imaging very diverse settings for all health care providers (e.g. emergency dept, pharmacy setting, etc etc).
This is another section where weight stigma/shame would seem to be very relevant.
Response: See response above.

Reviewer: 2

This is an interesting, organized, and well-written paper on weight control intentions and practices among adults in England and health professionals advice on this regard. Although the study objective needs to be refined and some references need to be updated, the methods are appropriate, and the discussion adequately represents the main results. The findings are not particularly novel, since weight loss attempts and the impact of health professional recommendations on these attempts have been previously explored in England (Piernas C et al, Int J Obes 2016; Jackson et al, BMJ Open 2013), but there is a need for more recent data. The authors should make this clearer in the introduction and strengths section. Also, I have a few suggestions that may be of use to further enhance the clarity and quality of the overall manuscript.

Title: I believe "Weight loss attempts and practices, and health professional advice in England: a cross-sectional national survey" is more reflective of the content of the manuscript.
Response: We have edited to: 'Weight control efforts and practices and health professional advice: a cross-sectional national survey in England'

Introduction:
(Page 2) The first paragraph needs to be reformulated/updated. There is a systematic review on weight loss attempts published in 2017 that includes more recent data than reference #1 and reports the strategies used around the world to try to lose weight. I believe reference #2 is not an obesity guideline, but a NHS weight loss plan/app. Proper obesity guidelines should be mentioned instead.
Response: We have updated the introduction to provide more recent data and appropriate references.

(Page 3, lines 27-31) The literature supports that women report more weight loss attempts than men – this should be reflected here.
Response: We now mention this:
'Evidence suggests community weight loss services are equally effective for men and women who attend,[11] but that men are less likely to be referred in the first place, which may reflect a bias amongst GPs or differences in patient demand, perhaps because some programmes are perceived as more suitable for women or because women make more weight loss attempts.[12]'

(Page 3, last paragraph) The stated purpose does not accord with the work that is presented – the authors are not testing impact of the health professional advice, but, instead, associations between advice and attempts. This need to be clarified here.
Response: We now provide a clearer statement of the study's aims and research questions.

Methods:
(Page 4, study design and sample) No need to repeat that data was collected in one month in October 2018. Authors should correct a typo: STS instead of SATS.
Response: We have made these changes.

Results:
(Pages 6-15) Some relative frequencies in the text don't match with relative frequencies in tables/figures (e.g., in Table 1 % of men and women; in Fig 3 % of eating healthily) – Authors

should cross-check the results section. In the text, the authors are mostly using people-first language regarding obesity, but not in Tables and Figures – this should be corrected (people with overweight/obesity instead of overweight/obese people).
Response: We have rerun all of the analyses and rewritten the results when revising the paper to ensure all errors and inconsistencies have been removed. We now use people-first language throughout.

(Page 6, line 56) State the BMI for obesity; also, BMI should be first mentioned as body mass index.
Response: We now define BMI as body mass index at first use in the introduction and provide BMI ranges for each weight status in the methods.

(Page 8, first paragraph) It seems that the authors are reporting comparisons between BMI categories instead of associations – this should be reframed; line 19: Please correct typo: "maintain weight after having lost weight" instead of "maintain weight having lost weight".
Response: We have made these changes.

Figures 1 and 2 can be merged in a bar chart instead of having two pie charts – it will facilitate the reading having bars for the entire sample and bars for the ones with a BMI above 30.
Response: We have removed the pie charts and now present all of the results in Tables.

(Page 10, lines 32-38) If there was no difference in the frequency of advice between men and women, how can one be more likely to be referred than the other? The authors can state a tendency, but only that. (Lines 50-53) Again, by how the results are stated, it seems between group comparisons and not odds ratios…
Response: We no longer discuss differences in receipt of advice between men and women.

Discussion:
Since weight loss attempts and the impact of health professional recommendations on these attempts have been previously explored in England and the novelty of this paper is having more recent data, the authors should reflect on how this may have changed since the last survey, although data may not have been collected in the same way.
Response: We now make the novelty of this study clearer in the discussion:
'Previous studies have documented positive associations between GP advice on weight loss and changes in patient weight management behaviours[8,9] and weight loss.[21] This study confirms and extends these findings, exploring associations between the type of advice received and different weight control practices. We found that people who recalled having received GP advice on weight loss were substantially more likely to report trying to lose weight.'

Strengths and limitations should be moved to the end of the discussion section.
Response: Done.

(Page 17) State all limitations together in the strengths and limitations section and not some of them within the comparisons with other literature section.
Response: Done.

(Pages 17-18, lines 53-4) If it wasn't statistically different the authors can't say it was greater…

Response: We have been more careful in our interpretation of non-significant results throughout the paper.

The entire text would benefit from attention to detail – typos, spaces between lines and words, parenthesis missing etc.
Response: We have tightened up the paper, paying attention to formatting and presentation.

PEER REVIEW HISTORY

BMJ Open publishes all reviews undertaken for accepted manuscripts. Reviewers are asked to complete a checklist review form and are provided with free text boxes to elaborate on their assessment. These free text comments are reproduced below.

ARTICLE DETAILS

Title (Provisional)

Weight control efforts and practices and health professional advice: a cross-sectional national survey in England

Authors

Jackson, Sarah; Warr, William; Brown, Jamie; Hartmann-Boyce, Jamie; Jebb, Susan A; Tudor, Kate; Shahab, Lion; Aveyard, Paul

---

VERSION 1 - REVIEW

**Reviewer**	**1**
**Name**	**Sturgiss, Elizabeth**
**Affiliation**	**Monash University, Department of General Practice**
**Date**	**01-Jul-2024**

COI **I am a GP-researcher with an academic interest in weight management in general practice. I have reviewed a previous version of this manuscript.**

Thank you for the opportunity to review the new version of this manuscript that outlines a survey of adults in England on their perception of GP weight management efforts.

I am not a statistician and have not reviewed the statistical methods of this paper.

Abstract

The objectives should make it clear that it is the perceptions of adult patients that are being examined, as it is currently written it sounds like it is file review or data review rather than self-report from patients.

Main manuscript

- I am still intrigued by the people in the healthy weight range who reported receiving weight loss advice; with 1% even saying they received a referral or medication; is this just pointing to the problems with self-reported weight/height and/or the problem of long-term recall for events? Page 14 still uses the total population of people who visited the GP as the denominator for those having recalled receiving weight loss advice - I don't understand why the denominator isn't those with overweight/obesity? I cannot think of any clinical guideline

that encourages weight loss advice to those in a healthy or underweight range. Lines 6-10 can probably be removed as the second paragraph makes more clinical sense.

- the limitations section is much improved and better reflects the methods of this work

- in the conclusion, "Only 1 in 5 thought health professionals discussing weight was inappropriate" - this is a high number, as per other parts of the manuscript and this sentence needs to reflect this.

The question that was asked in the survey, about health care providers rather than GPs, is a bit out of kilter with the rest of the survey that focused on GPs. This could be explored in the discussion. The fifth paragraph in the discussion is incorrect - the findings about this topic were about health care providers generally. I think that this paragraph would more helpfully explore the role of the therapeutic relationship - ie people are more comfortable discussing weight with a trusted GP, rather than any general health care provider. The therapeutic relationship also provides an alternate explanation for the vastly different result (<1%) in the BWeL trial who experienced the BI from their GP. I would suggest that this paragraph needs revision to better reflect the actual question that was asked in the survey.

**Reviewer**	**2**
**Name**	**Standen, Erin**
**Affiliation**	**Mayo Clinic**
**Date**	**11-Sep-2024**
**COI**	**I have no competing interests to declare.**

I am grateful for the opportunity to review the manuscript entitled, “Weight control efforts and practices and health professional advice: a cross-sectional national survey in England.” Although I did not review the original version of this manuscript, I see that the authors have substantially revised the paper based on an earlier round of reviewer feedback. I read through all enclosed materials, including the revised manuscript, STROBE checklist, and the authors’ responses to the editorial and reviewer comments. The manuscript is clear, well-written, and makes an important contribution to the literature about patient reactions to GPs’ weight-related advice. I have a few suggestions for how to strengthen the manuscript, most of which are related to how this evidence is situated and framed relative to the extant literature.

1. In the opening paragraph, the authors make the argument that because obesity rates have risen despite individuals being interested in weight loss, “existing intentions are not translating into effective action.” Certainly, there are individuals for whom this is the case (e.g., people who want to lose weight but take no or minimal action), but this statement is an oversimplification that places blame primarily on individuals rather than on the relative lack of effective intervention options. Meta-analytic evidence consistently indicates that

behavioral weight loss efforts (e.g., low-calorie diets, weight loss programs) rarely lead to lasting weight loss (i.e., substantial weight loss lasting for 1 year or longer; see Ge et al., 2020; Mann et al., 2007). Acknowledgment of the complex factors that determine body weight (e.g., genetics, hormones, environment) and the biological mechanisms that make prolonged weight loss difficult (e.g., metabolic adaptations to calorie restriction) would help contextualize the issue more accurately. Given that space is limited, another way to improve the opening paragraph would be to revise the statement to place any "blame" on the lack of accessible and effective interventions, rather than on individuals. For instance, "…obesity prevalence continues to increase, suggesting that further research is needed about how to connect people with effective interventions" - or something similar.

2. I was a bit confused by the claim that "In neither study were participants asked about the nature of advice, or their subsequent weight loss actions." Which two studies were being referred to? I ask because if this statement refers to the two references that precede it, one of them (Rose et al., 2013; #9 in the reference list) is a systematic review, and some of the studies included in it did ask about participants' subsequent weight loss actions. I imagine this may have come about due to re-arranging of sentences in a prior revision - citing the specific studies being referred to would address my concern.

3. I appreciate how clearly the research questions are stated; it makes this paper easy to follow and highlights its contributions effectively.

4. In Table 4, it might be helpful to mark, using bolding or asterisks, which ORs are statistically significant. Of course, that information is discernible from the 95% CIs, but an additional visual indicator would help readers focus on key information more quickly.

5. As some readers may be unfamiliar with the acronym "QOF," it may be helpful to provide the full program name.

6. Overall, the Discussion section fairly represents the results and limitations of this study, and I appreciate the acknowledgement of how things may have shifted post-COVID and in light of new GLP-1 medications. However, I think it is crucial for the Discussion to explicitly draw attention to the issue of weight bias/stigmatization of patients that occurs in healthcare (see Phelan et al., 2015 and Tomiyama et al., 2018, for reviews). Healthcare providers, including primary care physicians, are a major source of weight-based discrimination, and experiencing weight-based discrimination can make patients hesitant to return for future care. In other words, fear of experiencing weight stigma could explain why some of those 19% of participants do not want GPs to ask about their weight. Indeed, approximately 21% of participants reported that they were "neutral" about the appropriateness of GPs bringing up weight; and it is possible that members of this group have also experienced stigma from some GPs and therefore have mixed feelings about the practice. The authors do note that weight conversations should be destigmatized/normalized in primary care, but I think this misses the broader issue: that discussions of body weight can make patients feel stigmatized or belittled, particularly when GPs use language that patients

find offensive (see Puhl, 2020, for a review of stigmatizing weight terminology). Perhaps this section could be adjusted slightly to acknowledge the potential role of weight stigma and call for training for GPs to deliver obesity-related care that is both effective and stigma-free.

7. Finally, the framing "Only one in five thought health professionals discussing weight was inappropriate," gave me pause, at it minimizes this important group. 20% of the population is a large group of people, and disproportionately, folks who fell into that group were women and less socioeconomically advantaged. Earlier in the discussion, the authors called this a "substantial minority," which I think is more balanced. A simple, one-word way to address this might be to revise it to "Approximately one in five..."

VERSION 1 - AUTHOR RESPONSE

Reviewer: 1
Dr. Elizabeth Sturgiss, Monash University, The Australian National University

Thank you for the opportunity to review the new version of this manuscript that outlines a survey of adults in England on their perception of GP weight management efforts.
I am not a statistician and have not reviewed the statistical methods of this paper.

Abstract
The objectives should make it clear that it is the perceptions of adult patients that are being examined, as it is currently written it sounds like it is file review or data review rather than self-report from patients.
Response: We have edited the first sentence of this paragraph to make it clear data were self-reported:

> 'Using self-reported data collected as part of a nationally representative survey of adults in England…'

We have also edited research question 3 to clarify that we are talking about self-reports of receipt of GP advice, attempts to lose weight, and the type of advice received:

> 'To what extent is reporting receipt of GP advice on weight loss in the past year associated with reporting current attempts to lose weight and different weight management practices, and do these associations differ according to the type of advice people report receiving (specific referral or prescription medication for weight loss vs. general advice with no referral)?

Main manuscript
- I am still intrigued by the people in the healthy weight range who reported receiving weight loss advice; with 1% even saying they received a referral or medication; is this just pointing to the problems with self-reported weight/height and/or the problem of long-term recall for events? Page 14 still uses the total population of people who visited the GP as the denominator for those having recalled receiving weight loss advice - I don't understand why

the denominator isn't those with overweight/obesity? I cannot think of any clinical guideline that encourages weight loss advice to those in a healthy or underweight range. Lines 6-10 can probably be removed as the second paragraph makes more clinical sense.

Response: The reason we included all participants who had visited their GP in this analysis is because the time frame used in the questions means we could not determine participants' BMI at the time they received GP advice on weight loss. The questions assessing BMI referred to participants' current status at the time of the survey, while receipt of GP advice referred to the last 12 months. As such, it is plausible that some participants may have only had a healthy BMI at the time of the survey after having received GP advice and losing weight; excluding them from the analysis may therefore lead us to underestimate the proportion receiving advice. We have added clarification in the Methods as to why we included all participants in this analysis:

> 'We estimated the prevalence of receipt of advice among all participants who had visited their GP in the last year and specifically among those with a BMI in the overweight or obesity range at the time of the survey. While the former group included participants with a BMI in the healthy weight range, it is plausible that some of these participants may have had a higher BMI, received GP advice on weight loss, and subsequently lost weight (as the question asked about receipt of advice over the past year).'

As suggested, we have removed from the results text the sentences referring to receipt of GP advice among all participants (leaving this information in table 3). Instead, the results text now focuses on those with a BMI in the overweight or obesity range:

> 'Of participants, 68.6% (n=1,179) reported having visited their GP in the last 12 months. Among those with a BMI in the overweight or obesity range, 12.6% and 40.0% respectively recalled having received advice on weight loss (Table 3). Among both groups, this was largely general advice (10.2% and 30.8%, respectively): just 2.4% of those with overweight and 9.2% of those with obesity reported receiving a referral or prescription for weight-loss medication.'

We have updated research question 2 to reflect this focus on those with overweight or obesity specifically:

> 'Among those who have visited their GP in the last 12 months (and specifically among those with a BMI in the overweight or obesity range at the time of the survey), what proportion report having received advice on weight loss in the past year and what was the nature of this advice?'

We also refer to the limitation of the timing of questions in the discussion, and now suggest a potential explanation for the few participants with underweight BMIs reporting having received GP advice on weight loss:

> 'Thirdly, there may be some misclassification: BMI data were based on current self-reported weight at time of survey, but the medical advice could have been received at any time during the past 12 months. Therefore, it is possible that some participants may have received advice while having overweight or obesity and

subsequently lost weight, meaning their weight status had changed by the time they were surveyed. A few participants with an underweight BMI reported having received GP advice on weight loss; it is likely this was not advice to lose weight but may have been advice on how to reverse or prevent further weight loss. It is also possible that GPs provided advice incorrectly due to misclassification or participants mistakenly recalled having received such advice.'

- the limitations section is much improved and better reflects the methods of this work

Response: Thank you.

- in the conclusion, "Only 1 in 5 thought health professionals discussing weight was inappropriate" - this is a high number, as per other parts of the manuscript and this sentence needs to reflect this.

Response: We have edited to 'Approximately one in five…'

The question that was asked in the survey, about health care providers rather than GPs, is a bit out of kilter with the rest of the survey that focused on GPs. This could be explored in the discussion. The fifth paragraph in the discussion is incorrect - the findings about this topic were about health care providers generally. I think that this paragraph would more helpfully explore the role of the therapeutic relationship - ie people are more comfortable discussing weight with a trusted GP, rather than any general health care provider. The therapeutic relationship also provides an alternate explanation for the vastly different result (<1%) in the BWeL trial who experienced the BI from their GP. I would suggest that this paragraph needs revision to better reflect the actual question that was asked in the survey.

Response: We have corrected the first sentence of this paragraph so that it matches the question asked (i.e,. refers to health care providers rather than GPs):

> 'Our findings show that while the general population are broadly supportive of weight interventions by health care providers, a substantial minority are not'

We have also reworked the paragraph to include discussion about the question asking about health care providers rather than GPs specifically, drawing on the reviewer's suggestions:

> 'This finding may partly reflect the wording of the question referring to 'health care providers' in general, as opposed to GPs specifically: people may be more comfortable discussing weight with a trusted GP, rather than any health professional more generally. Experience from the BWeL trial shows that almost none of the patients (<1%) who received an opportunistic intervention by trained GPs found it inappropriate and unhelpful.[6] However, people being uncomfortable with health professionals discussing weight may also be linked to weight bias and stigmatisation they have experienced in health care.[26,27] Patients with obesity are sensitive to cues that the doctor is judging them negatively and may be sensitised to this from experience of flippant weight loss advice.[17] Discussions about weight can make patients feel stigmatised and belittled, particularly when health care providers use

language that patients find offensive.[28] Fear of experiencing weight stigma could therefore be a reason why some participants did not want health professionals to discuss their weight. Specific GP training to initiate sensitive, effective, and non-stigmatising weight management interventions could reduce patient resistance.'

Reviewer: 2

Dr. Eric Standen, Mayo Clinic

I am grateful for the opportunity to review the manuscript entitled, "Weight control efforts and practices and health professional advice: a cross-sectional national survey in England." Although I did not review the original version of this manuscript, I see that the authors have substantially revised the paper based on an earlier round of reviewer feedback. I read through all enclosed materials, including the revised manuscript, STROBE checklist, and the authors' responses to the editorial and reviewer comments. The manuscript is clear, well-written, and makes an important contribution to the literature about patient reactions to GPs' weight-related advice. I have a few suggestions for how to strengthen the manuscript, most of which are related to how this evidence is situated and framed relative to the extant literature.

1. In the opening paragraph, the authors make the argument that because obesity rates have risen despite individuals being interested in weight loss, "existing intentions are not translating into effective action." Certainly, there are individuals for whom this is the case (e.g., people who want to lose weight but take no or minimal action), but this statement is an oversimplification that places blame primarily on individuals rather than on the relative lack of effective intervention options. Meta-analytic evidence consistently indicates that behavioral weight loss efforts (e.g., low-calorie diets, weight loss programs) rarely lead to lasting weight loss (i.e., substantial weight loss lasting for 1 year or longer; see Ge et al., 2020; Mann et al., 2007). Acknowledgment of the complex factors that determine body weight (e.g., genetics, hormones, environment) and the biological mechanisms that make prolonged weight loss difficult (e.g., metabolic adaptations to calorie restriction) would help contextualize the issue more accurately. Given that space is limited, another way to improve the opening paragraph would be to revise the statement to place any "blame" on the lack of accessible and effective interventions, rather than on individuals. For instance, "…obesity prevalence continues to increase, suggesting that further research is needed about how to connect people with effective interventions" - or something similar.
Response: We appreciate this point, and the suggested alternative wording. We have edited the sentence to read:

> 'Despite a strong desire to lose weight among the majority of adults with obesity[4] and a substantial weight loss industry, obesity prevalence continues to increase,[5] suggesting further research is needed about how to connect people with effective interventions.'

2. I was a bit confused by the claim that "In neither study were participants asked about the nature of advice, or their subsequent weight loss actions." Which two studies were being referred to? I ask because if this statement refers to the two references that precede it, one of them (Rose et al., 2013; #9 in the reference list) is a systematic review, and some of the studies included in it did ask about participants' subsequent weight loss actions. I imagine this may have come about due to re-arranging of sentences in a prior revision - citing the specific studies being referred to would address my concern.
Response: Thank you for drawing our attention to this – it was indeed a mistake made in revising the paper. We have edited this sentence so that it now refers only to the latter of the two studies (i.e., excludes the systematic review):

> 'However, participants were not asked about the nature of advice, or their subsequent weight loss actions.[7]'

3. I appreciate how clearly the research questions are stated; it makes this paper easy to follow and highlights its contributions effectively.
Response: Thank you.

4. In Table 4, it might be helpful to mark, using bolding or asterisks, which ORs are statistically significant. Of course, that information is discernible from the 95% CIs, but an additional visual indicator would help readers focus on key information more quickly.
Response: Done.

5. As some readers may be unfamiliar with the acronym "QOF," it may be helpful to provide the full program name.
Response: Done – this sentence now reads:

> 'This study provides further evidence of the need to introduce policies to encourage implementation of the clinical guidelines, including considering measures that have been effective at changing GP behaviours in other areas, such as Quality and Outcomes Framework (QOF) incentivisation or Enhanced Service Payments'

6. Overall, the Discussion section fairly represents the results and limitations of this study, and I appreciate the acknowledgement of how things may have shifted post-COVID and in light of new GLP-1 medications. However, I think it is crucial for the Discussion to explicitly draw attention to the issue of weight bias/stigmatization of patients that occurs in healthcare (see Phelan et al., 2015 and Tomiyama et al., 2018, for reviews). Healthcare providers, including primary care physicians, are a major source of weight-based discrimination, and experiencing weight-based discrimination can make patients hesitant to return for future care. In other words, fear of experiencing weight stigma could explain why some of those 19% of participants do not want GPs to ask about their weight. Indeed, approximately 21% of participants reported that they were "neutral" about the

appropriateness of GPs bringing up weight; and it is possible that members of this group have also experienced stigma from some GPs and therefore have mixed feelings about the practice. The authors do note that weight conversations should be destigmatized/normalized in primary care, but I think this misses the broader issue: that discussions of body weight can make patients feel stigmatized or belittled, particularly when GPs use language that patients find offensive (see Puhl, 2020, for a review of stigmatizing weight terminology). Perhaps this section could be adjusted slightly to acknowledge the potential role of weight stigma and call for training for GPs to deliver obesity-related care that is both effective and stigma-free.
Response: We have edited this paragraph to incorporate these excellent suggestions. It now reads:

> 'Our findings show that while the general population are broadly supportive of weight interventions by health care providers, a substantial minority are not: 19% thought that health professionals should not ask about weight. This finding may partly reflect the wording of the question referring to 'health care providers' in general, as opposed to GPs specifically: people may be more comfortable discussing weight with a trusted GP, rather than any health professional more generally. Experience from the BWeL trial shows that almost none of the patients (<1%) who received an opportunistic intervention by trained GPs found it inappropriate and unhelpful.[6] However, people being uncomfortable with health professionals discussing weight may also be linked to weight bias and stigmatisation they have experienced in health care.[26,27] Patients with obesity are sensitive to cues that the doctor is judging them negatively and may be sensitised to this from experience of flippant weight loss advice.[17] Discussions about weight can make patients feel stigmatised and belittled, particularly when health care providers use language that patients find offensive.[28] Fear of experiencing weight stigma could therefore be a reason why some participants did not want health professionals to discuss their weight. Specific GP training to initiate sensitive, effective, and non-stigmatising weight management interventions could reduce patient resistance.'

7. Finally, the framing "Only one in five thought health professionals discussing weight was inappropriate," gave me pause, at it minimizes this important group. 20% of the population is a large group of people, and disproportionately, folks who fell into that group were women and less socioeconomically advantaged. Earlier in the discussion, the authors called this a "substantial minority," which I think is more balanced. A simple, one-word way to address this might be to revise it to "Approximately one in five..."
Response: We have edited as suggested to 'Approximately one in five…'

VERSION 2 - REVIEW

**Reviewer**	**2**
**Name**	**Standen, Erin**
**Affiliation**	**Mayo Clinic**
**Date**	**16-Oct-2024**
**COI**	

The authors have addressed all of my comments appropriately, and I am happy to see the thoughtful paragraph that has been added about the role of weight stigma in patients' experiences of receiving weight loss advice from a GP. I also appreciate the additional discussion of the study's limitations.
